# Circulating *MicroRNA-92b-3p* as a Novel Biomarker for Monitoring of Synovial Sarcoma

**DOI:** 10.1038/s41598-017-12660-5

**Published:** 2017-11-07

**Authors:** Koji Uotani, Tomohiro Fujiwara, Aki Yoshida, Shintaro Iwata, Takuya Morita, Masahiro Kiyono, Suguru Yokoo, Toshiyuki Kunisada, Ken Takeda, Joe Hasei, Kunihiko Numoto, Yutaka Nezu, Tsukasa Yonemoto, Takeshi Ishii, Akira Kawai, Takahiro Ochiya, Toshifumi Ozaki

**Affiliations:** 1 Department of Orthopaedic Surgery, Okayama University Graduate School of Medicine, Dentistry, and Pharmaceutical Sciences, Okayama, Japan; 2 Center of Innovative Medicine, Okayama University Hospital, Okayama, Japan; 3 Department of Orthopaedic Surgery, Chiba Cancer Center, Okayama, Japan; 4 Department of Medical Materials for Musculoskeletal Reconstruction, Okayama University Graduate School of Medicine, Dentistry, and Pharmaceutical Sciences, Okayama, Japan; 5 Department of Intelligent Orthopaedic System, Okayama University Graduate School of Medicine, Dentistry, and Pharmaceutical Sciences, Okayama, Japan; 6 Department of Orthopaedic Surgery, Kochi Health Sciences Center, Kochi, Japan; 7 Division of Molecular and Cellular Medicine, National Cancer Center Research Institute, Tokyo, Japan; 8 Department of Musculoskeletal Oncology, National Cancer Center Hospital, Tokyo, Japan

## Abstract

The lack of useful biomarkers is a crucial problem for patients with soft tissue sarcomas (STSs). Emerging evidence has suggested that circulating microRNAs (miRNAs) in body fluids have novel impact as biomarkers for patients with malignant diseases, but their significance in synovial sarcoma (SS) patients remains unknown. Initial global miRNA screening using SS patient serum and SS cell culture media identified a signature of four upregulated miRNAs. Among these candidates, *miR-92b-3p* secretion from SS cells was confirmed, which was embedded within tumour-derived exosomes rather than argonaute-2. Animal experiments revealed a close correlation between serum *miR-92b-3p* levels and tumour dynamics. Clinical relevance was validated in two independent clinical cohorts, and we subsequently identified that serum *miR-92b-3p* levels were significantly higher in SS patients in comparison to that in healthy individuals. Moreover, serum *miR-92b-3p* was robust in discriminating patients with SS from the other STS patients and reflected tumour burden in SS patients. Overall, liquid biopsy using serum *miR-92b-3p* expression levels may represent a novel approach for monitoring tumour dynamics of SS.

## Introduction

Synovial sarcoma (SS) is a high-grade soft tissue sarcoma (STS) that accounts for 10% to 20% of STSs in adolescents and in the young adult population^1,2^. The incidence is estimated to be 2 per 100,000 people^3^. Reported 5-year survival rates for SS range from 36% to 76% and reported 10-year survival rates range from 20% to 63%^4,5^. The difference in the 5- and 10-year survival rates reflects the relatively high incidence of late metastases. Metastatic lesions develop in about half of cases, most commonly to the lung, followed by the lymph nodes and the bone marrow. With adequate surgical excision or with adjunctive therapy, the recurrence rate has been reported to be less than 40%. In most cases the recurrent growth manifests within the first 2 years after initial therapy^3^. Since the outcomes are far worse for SS patients who present with local recurrence or metastasis, the early diagnosis of tumour, recurrence, metastasis, and even drug response is crucial for better management. Indeed, this tumour is characterized by the *SS18-SSX* fusion gene, and the presence of this chromosomal translocation is clinically useful as a diagnostic marker. However, it does not reflect disease progression and is only evaluated using tumour specimens surgically resected^6^. To date, there are no useful non-invasive biomarkers for tumour monitoring of SS.

microRNAs (miRNAs) are small non-coding RNAs involved in post-transcriptional regulation of gene expression in the cytoplasm, and they can influence a variety of biological processes, including development, proliferation and differentiation^7^. Accumulating evidence indicates that miRNAs may function as either tumour suppressors or oncogenes that regulates growth and apoptosis^7^. Recent reports have demonstrated that they exist with remarkable stability in body fluids as cell-free miRNA that originates from primary tumour cells embedded within tumour-derived exosomes or argonatute-2 (Ago2). Circulating cell-free miRNA is attracting attention as a target of liquid biopsy, including as circulating cell-free DNA or in circulating tumour cells^8–12^. While evidence on circulating miRNAs has been accumulated in various cancers, there has been little involving the soft tissue sarcomas, which to date lack useful circulating biomarkers.

We investigated the expression profiles of serum cell-free miRNAs using blood samples from SS patients as well as in other STS patients, compared to controls, followed by the evaluation of biological and clinical significance using *in vitro* and *in vivo* experimental procedures and involving independent patient cohorts.

## Results

### Global miRNA microarray profiling of SS patient serum and SS cell culture media

**Figure 1:**
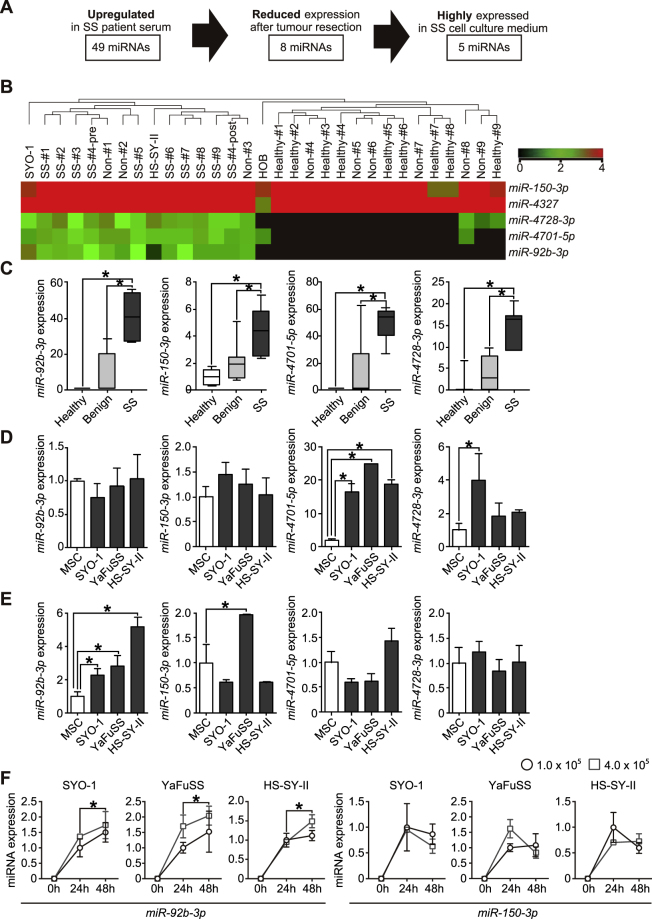
Identification and experimental validation of circulating/secretory miRNAs in SS-patient serum. (**A**) Schematic representation of the approach used for the detection of circulating/secretory miRNAs in SS-patients. (**B**) Heatmap and the hierarchical clustering of miRNA microarray analysis using patient serum in preoperative state as well as in cell culture media of SS cells. (**C**) Serum levels of the candidate markers in SS patients, age-matched benign tumour patients, and healthy individuals. *p < 0.05; Student’s *t* test. (**D**) Expression levels of candidate miRNAs in SS cells and human mesenchymal stem cells (hMSCs). Data are presented as mean ± S.D. (n = 3 in each group) *p < 0.05; Student’s *t* test. (**E**) Expression levels of candidate miRNA biomarkers in culture media of SS cells (SYO-1, YaFuSS, and HS-SY-II) and hMSCs. Data are presented as mean ± S.D. (n = 3 in each group) *p < 0.05; Student’s *t* test. (**F**) The expression dynamics of *miR-92b-3p* and *miR-150-3p* in the culture media of SS cell lines according to cell number and the culture duration. Data are presented as mean ± S.D. (n = 3 in each group) *p < 0.05; Student’s *t* test.

Microarray profiling analysis was performed on nine pairs of serum samples obtained from SS-patients, age-matched benign tumour patients, and from healthy individuals, as well as culture media of SYO-1 and HS-SY-II SS cell lines. Characteristics of this cohort are shown in Supplementary Tables S1 and S2. Following hierarchical clustering, candidates were narrowed down to the upregulated miRNAs (fold change >1.5) with statistical significance (*p* < 0.05). Forty-nine serum miRNAs were significantly upregulated in SS patients compared with controls, and eight among these 49 miRNAs were markedly reduced at post-operative status compared to pre-operative status. Of the eight miRNAs, 5 were also highly expressed in culture media of SS cell lines (Fig. 1A,B). Then, *miR-92b-3p*, *miR-150-3p*, *miR-4701-5p* and *miR-4728-3p*, for which qPCR reporter probes were available, were selected for the further detailed analysis. Each of these candidates was highly expressed in SS patients compared to controls (Fig. 1C).

### Identification of miRNAs secreted from SS cells

The miRNA candidates were analyzed for their expression levels in the tumour cells and in the culture media of SYO-1, HS-SY-II, and YaFuSS cell lines. RT-qPCR revealed that these candidates were evidently expressed in all SS cell lines, and *miR-4701-5p* expression was significantly upregulated in all SS cells compared with that observed in control hMSCs (*p* < 0.05, Fig. 1D). On the other hand, *miR-92b-3p* expression in the culture media of all SS cell lines examined and *miR-150-3p* expression in the culture medium of YaFuSS was significantly higher than that of hMSCs (*p* < 0.05, Fig. 1E), suggesting that *miR-92b-3p* and *miR-150-3p* are abundantly secreted from SS-cells.

Next, the secretion of these candidate miRNAs from SS cells was evaluated using established SS cell lines. Expression levels of *miR-92b-3p* in culture media of each SS cell line increased with the number of tumour cells and duration of the incubation, whereas *miR-150-3p* expression levels in culture media did not correlate with these measures (Fig. 1F), indicating that *miR-92b-3p* is clearly secreted from SS-cells.

### Serial monitoring of miRNAs in SS tumour-bearing mice

**Figure 2:**
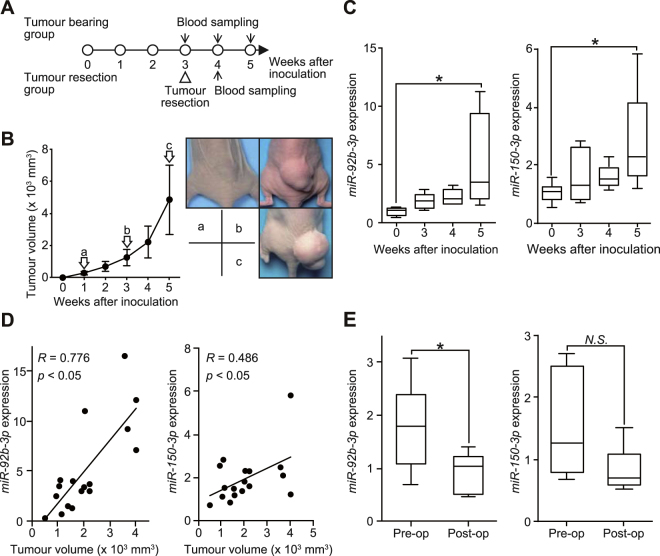
Serum expression levels of candidate miRNAs during tumour development in SS-bearing mice. (**A**) Scheme of the animal experiment. (**B**) The tumour volume plotted each week after tumour inoculation (left). The macroscopic appearance of SYO-1 tumours in each group of mice 1, 3 and 5 weeks after tumour injection are shown (right). (**C**) Serum *miR-92b-3p* and *miR-150-3p* expression at 0, 3, 4, and 5 weeks after tumour inoculation. *p < 0.05; Student’s *t* test (**D**) Pearson’s correlation between *miR-92b-3p* and *miR-150-3p* levels and tumour volume (*R* = 0.776 for *miR-92b-3p*, *R* = 0.486 for *miR-150-3p*, *p* < 0.05, respectively). (**E**) Relative serum *miR-92b-3p* and *miR-150-3p* levels at pre- and postoperative stages (n = 6 in each group) *p < 0.05; Student’s *t* test.

To evaluate whether serum *miR-92b-3p* levels could be used to monitor tumour dynamics *in vivo*, we evaluated possible correlations between tumour growth and serum *miR-92b-3p* expression levels using SYO-1-bearing mice (Fig. 2A). After subcutaneous transplantation of SYO-1 cells into mouse hind quarters, serum *miR-92b-3p* and *miR-150-3p* levels were investigated and their identified elevation in association with the growing tumour volume was established (Fig. 2B,C). We observed statistical significance between tumour size and serum *miR-92b-3p* levels (*R* = 0.776, *p* < 0.05), while serum *miR-150-3p* levels were partially correlated with tumour growth (*R* = 0.486, *p* < 0.05, Fig. 2D). Furthermore, serum *miR-92b-3p* levels significantly decreased after tumour resection (Fig. 2E). These results suggested that serum levels of *miR-92b-3p*, rather than of *miR-150-3p*, reflect tumour burden in SYO-1-bearing mice.

### Correlation of serum *miR-92b-3p* with tumour burden in SS patients

**Figure 3:**
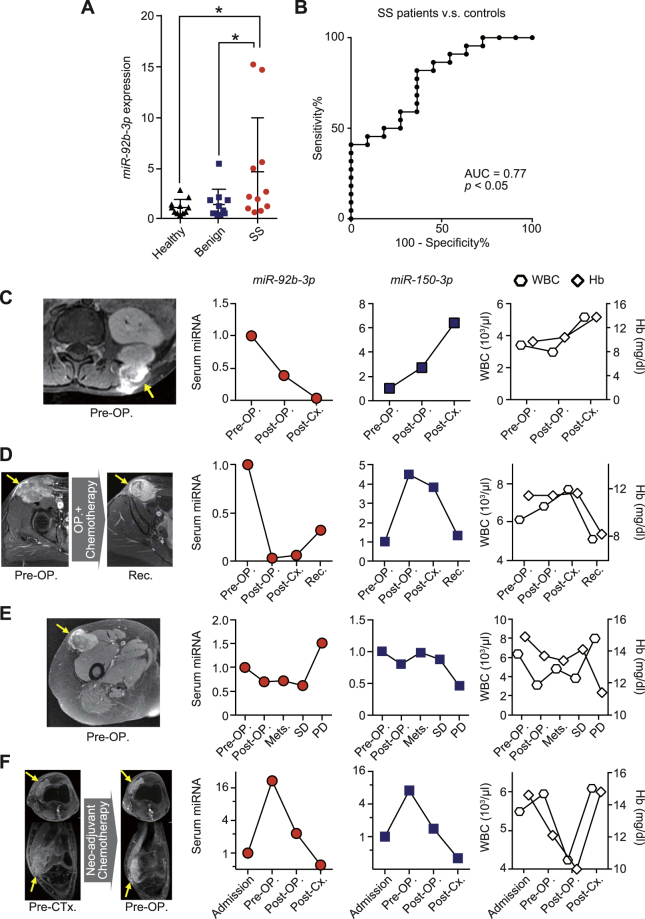
Serum *miR-92b-3p* expression levels and dynamics in synovial sarcoma patients in the validation cohort. (**A**) Serum *miR-92b-3p* expression levels in SS patients and control individuals in the validation cohort (*p* < 0.05) *p < 0.05; one-way ANOVA with Holm-Sidak’s multiple comparison test. (**B**) Receiver operating characteristic (ROC) curve analysis. ROC curve analysis indicated the AUC of 0.77 (95% confidence interval = 0.61–0.94) discriminating SS from age-matched benign tumour patients and healthy individuals. (**C**–**F**) Tumour monitoring of serum *miR-92b-3p* levels during multimodal therapies. Four SS patients including an 11 year old male with lower back involvement (**C**), 39 year old female (groin region) (**D**), 61 year old female (proximal thigh) (**E**), and 21 year old male (knee joint) (**F**) could be evaluated during the treatment. Abbreviations: WBC = white blood cell; Hb = hemoglobin; OP = operative surgery; Cx = chemotherapy; Rec = recurrence; Mets = metastasis; SD = stable disease; PD = progressive disease.

Next, we analyzed the serum *miR-92b-3p* and *miR-150-3p* levels in a validation cohort of SS patients, age-matched benign tumour patients, and healthy individuals (n = 12, each). The demographics and clinical characteristics of patients and healthy individuals of the validation cohort are described in Supplementary Table S1 and S2. There were no significant differences in age or gender between groups. The expression levels of serum *miR-92b-3p* were significantly higher in SS patients than in age-matched benign tumour patients and healthy individuals (*p* < 0.05, Fig. 3A). Receiver operation characteristic (ROC) analysis revealed that serum *miR-92b-3p* levels contributed to the capacity to distinguish patients with SS from age-matched controls and healthy individuals, with area under the curve (AUC) value of 0.77 (95% confidence interval (CI) = 0.61–0.94) (Fig. 3B). The sensitivity and specificity of serum *miR-92b-3p* levels were 81.8% and 63.6%, respectively. On the other hand, the AUC value of ROC analysis based on serum *miR-150-3p* levels was 0.94 (95% CI = 0.86–1.0) for control individuals (Supplementary Figure 1A and B). Although there was no correlation with the expression levels of both *miR-92b-3p* and *miR-150-3p* or age, gender, tumour location, and presence of lung metastasis at diagnosis, univariate analysis demonstrated that serum *miR-92b-3p* tended to correlate with tumour size (Supplementary Table S3).

### Serum *miR-92b-3p* expression levels for clinical tumour monitoring

To further investigate the clinical utility of serum *miR-92b-3p* for tumour monitoring, we evaluated serum *miR-92b-3p* and *miR-150-3p* levels, as well as white blood cell (WBC) counts and hemoglobin (Hb) levels in SS patients from whom we could obtain a series of serum samples during multimodal treatment. Relative expression levels of miRNAs were evaluated by standardization to the initial expression levels of each subject. Case 1 was an 11 year-old male with SS arising in his lower back (Fig. 3C). Serum *miR-92b-3p* levels decreased after tumour resection and adjuvant chemotherapy, whereas serum *miR-150-3p* levels did not. Case 2 was a 39 year-old female with SS arising in the groin region (Fig. 3D). Serum *miR-92b-3p* levels decreased after tumour resection and adjuvant chemotherapy, but gradually increased after local recurrence, whereas serum *miR-150-3p* levels did not reflect tumour dynamics. Case 3 was a 61 year-old female with SS in her proximal thigh, with lung metastasis at diagnosis (Fig. 3E). Serum *miR-92b-3p* levels decreased after tumour resection, and slightly increased with the growth of the lung metastasis, and finally exhibited further increase with progressive disease. In contrast, serum *miR-150-3p* levels did not correlate with disease progression. Case 4 involved a 21 year-old male with SS in his knee joint (Fig. 3F). In this instance, the response to neo-adjuvant chemotherapy based on adriamycin and ifosfamide was poor. Serum levels of *miR-92b-3p* and *miR-150-3p* increased after neo-adjuvant chemotherapy, but these parameters decreased gradually after tumour resection and adjuvant chemotherapy by gemcitabine and docetaxel. In all cases, measures of WBC and Hb did not correlate with *miR-92b-3p* levels, suggesting that this miRNA was not secreted from hematocytes. Overall, serum *miR-92b-3p* levels could be useful for tumour monitoring in SS patients.

### Secreted *miR-92b-3p* expression levels from SS and other soft tissue sarcoma cells

**Figure 4:**
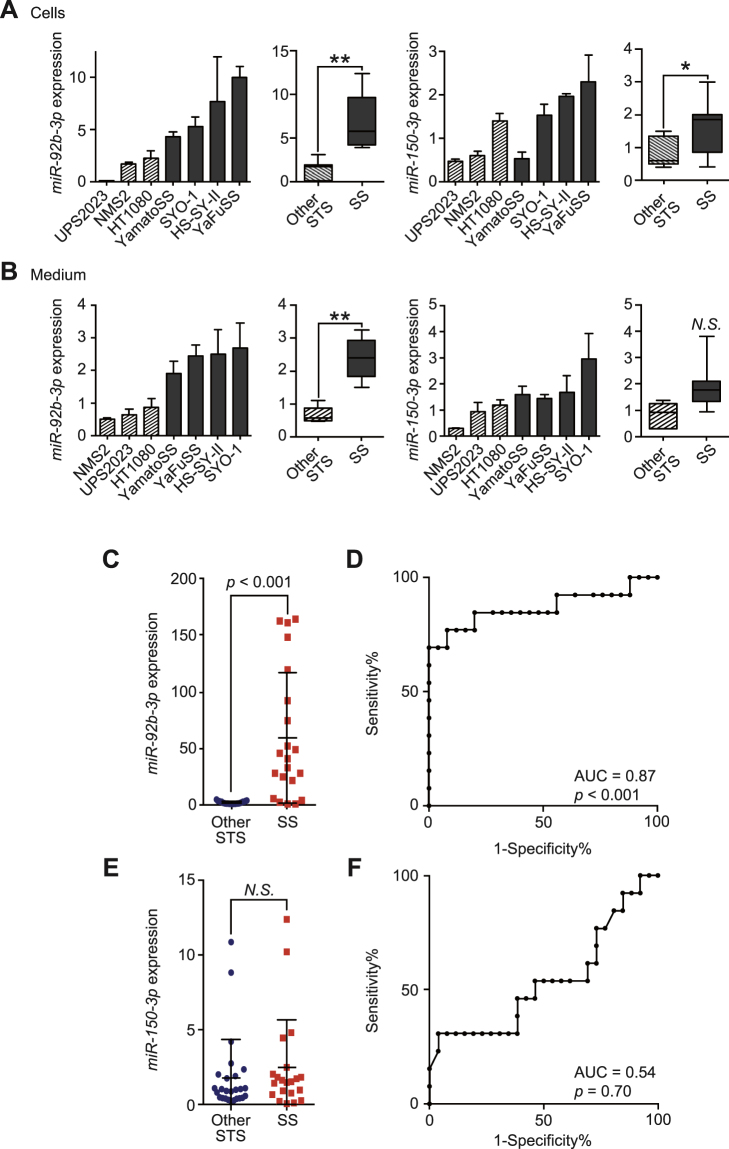
Differential diagnosis of synovial sarcoma from other soft tissue sarcomas by circulating/secreted *miR-92b-3p*. (**A**,**B**) The expression of *miR-92b-3p* and *miR-150-3p* in cells (**A**) and cell culture media (**B**) of SS (SYO-1, YaFuSS, HS-SY-II, and Yamato-SS) and the other STS cell lines (HT1080, NMS2, and UPS2023). The Mann-Whitney *U* test was used for comparison between groups. (**C**) Serum *miR-92b-3p* levels in SS patients and other STS patients (*p* < 0.001). The Mann-Whitney *U* test was used for comparison between groups. (**D**) ROC curve analysis of *miR-92b-3p* discriminating SS patients from other STS patients. AUC was 0.88 (95% confidence interval = 0.72–1.0). (**E**) Serum *miR-150-3p* expression levels in SS patients and other STS patients. The Mann-Whitney *U* test was used for comparison between groups. (**F**) ROC curve analysis of *miR-150-3p* discriminating SS patients from other STS patients. AUC was 0.54 (95% confidence interval = 0.33–0.75).

To investigate whether cell-free *miR-92b-3p* was specifically secreted from SS cells compared to other STS cell types, we evaluated expression levels of *miR-92b-3p* in culture media of SS and other STS cell lines described. We identified that *miR-92b-3p* expression levels in cells and culture media were significantly higher in SS cells than in other STS cells (*p* < 0.001, Fig. 4A,B). On the other hand, *miR-150-3p* expression levels were also significantly higher in SS cells but did not show statistically significant difference in culture media of SS and other STS cell lines described (Fig. 4A,B).

### Differential diagnosis of SS and other STS patients by serum *miR-92b-3p* quantification

To assess whether quantification of serum *miR-92b-3p* levels could be used for differential diagnosis, we examined levels in patients with SS (n = 12) and other STS patients (n = 24). Demographics and histological classification of the other STS patients are described in Supplementary Table S4. Serum *miR-92b-3p* levels demonstrated a significant upregulation in SS patients compared with the other STS patients at time of diagnosis (*p* < 0.0001, Fig. 4C). ROC curve depiction reveals the diagnostic significance of serum *miR-92b-3p* quantification differentiating SS from other STS with AUC value of 0.87 (*p* < 0.0001, 95% CI = 0.72–1.0, Fig. 4D). ROC curve analysis revealed sensitivity and specificity of *miR-92b-3p* determination as 84.6% and 80.0%, respectively. In contrast, the expression levels of serum *miR-150-3p* did not reveal any significant difference between SS and the other STS patients, and AUC numerics were poor (Fig. 4E,F).

### Secreted *miR-92b-3p* in tumour-derived exosomes

**Figure 5:**
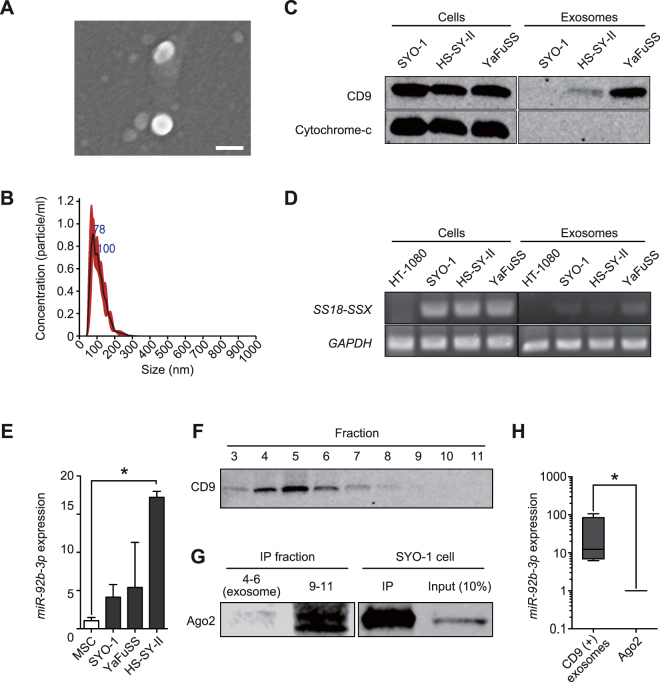
*miR-92b-3p* secretion with tumour-derived exosomes. (**A**) Scanning electron microscope image of purified exosomes, which were essentially homogeneous at 40–200 nm in diameter. Bar: 100 nm. (**B**) The size distribution of exosomes assessed by the NanoSight^®^ nanoparticle tracking system. The size range of isolated exosomes was approximately 50–200 nm, peaking at 100 nm. (**C**) Western blotting of the exosomes derived from SS (SYO-1, HS-SY-II, YaFuSS). Staining for tetraspanin protein (CD9, 25 kDa) was positive for both cell lysates and exosomes, whereas probing for cytochrome-c (15 kDa) was negative for exosomes. Full-length blots are presented in Supplementary Figure S2. (**D**) Polymerase chain reaction of *SS18-SSX* fusion gene. *SS18-SSX* was detected for both cells and exosomes of cultured SS cells (SYO-1, HS-SY-II, YaFuSS). HT1080 fibrosarcoma cell line was used as negative control. Full-length gels are presented in Supplementary Figure S3. (**E**) *miR-92b-3p* expression levels in SS-derived exosomes. hMSCs was used as a negative control *p < 0.05; Student’s *t* test. (**F**) Western blotting of SS patient serum for each fraction of EV-second^®^ procedure. Fraction 3 to 7 were positive for CD9 (25 kDa). Strongly positive fractions 4 to 6 mainly contain exosomes. Full-length blots are presented in Supplementary Figure S4. (**G**) Fractions containing exosomes (fractions 4 to 6) and subsequent fractions (fractions 9 to 11), which contain larger-sized proteins than earlier fractions, which were immunoprecipitated using human anti-Ago2 monoclonal antibody. SYO-1 was used as a positive control. Western blotting revealed Ago2 (100 kDa) was negative in exosome fractions and positive for fractions 9 to 11, suggesting these fractions mainly contain Ago2. Full-length blots are presented in Supplementary Figure S5. (**H**) The expression of *miR-92b-3p* in exosomes and Ago2 concentrations *p < 0.05; Mann-Whitney *U* test.

To investigate the basis of *miR-92b-3p* stability in the extracellular environment, we evaluated levels of this miRNA in exosomes derived from SS cell lines. The collected exosomes were identified using SEM as essentially homogeneous vesicles of 40–200 nm in diameter (Fig. 5A), which was confirmed by employing the NS300 NanoSight^®^ (Fig. 5B). Western blotting revealed that the isolated exosomes were positive for CD9 and negative for cytochrome-c (Fig. 5C) and the *SS18-SSX* fusion gene was separately detected in the tumour-derived exosomes (Fig. 5D). Moreover, the expression levels of *miR-92b-3p* were higher in exosomes derived from SS cells than in hMSCs (Fig. 5E). These results suggested that *miR-92b-3p* is loaded in exosomes derived from SS cells.

### Differential expression of *miR-92b-3p* in exosomes and Ago2 derived from SS-patient serum

To further identify how *miR-92b-3p* is stable during circulation in SS patients, we evaluated *miR-92b-3p* levels in both exosomes and Ago2 from sera of SS patients. The serum samples were fractionated by using EV-second^®^, followed by IP with anti-Ago2 antibody. CD9 expression differentiated the exosomes from Ago2-positive fractions, which was confirmed by western blot analysis (Fig. 5F,G). Our investigations identified that *miR-92b-3p* levels were significantly elevated in exosomes than in Ago2-positive fractions of SS-patient serum (Fig. 5H), indicating that this miRNA circulates in SS patients and is loaded on tumour-derived exosomes. Although we additionally evaluated serum *SS18-SSX* fusion gene transcript, it was not detected in SS-patient serum or exosome fractions within the serum (Supplementary Figure S6 and Supplementary Tables S5, 6). These observations offer immediate tumour surveillance and future potential therapeutic avenues.

## Discussion

To date, miRNA dysregulation in SS cells or tissue specimens has been reported by several groups (Supplementary Table S7)^13–18^. The representative functional miRNAs in SS cells includes the upregulated *miR-17-5p* ^18^, *miR-99b*, *miR-125a* ^15^, *miR-183* ^17^, and the downregulated *miR-143* ^16^. Our global miRNA profiling analysis using SS patient serum and SS cell culture media demonstrated dissimilar patterns, compared to the reported miRNA dysregulation in SS cells, and *miR-92b-3p* has never been identified. Similarly, several investigators have also demonstrated the dissimilarity between cellular miRNAs and secreted miRNAs in various cancers, such as breast cancer^19–22^. These dissimilarities in the miRNA expression pattern may indicate the existence of molecular mechanisms regulating secretion of miRNAs, which has been suggested by several researchers^23,24^. Therefore, our approach of global miRNA analysis based on patient blood samples, rather than focusing on dysregulated miRNA within tumour cells or tissue specimens, could be a suitable method for investigation of clinically important circulating miRNA. Importantly, researchers have to pay attention to the evidence that a variety of circulating miRNAs, reported as circulating cancer biomarkers, reflect a secondary effect on patient blood cells rather than a tumour cell-specific origin^25^. *miR-92b-3p* has not been reported as a hematocyte-derived miRNA and we confirmed here that hematocytes were not associated with serum *miR-92b-3p* levels.

Recent investigations have demonstrated that exosomes are enriched in bioactive molecules, contain nucleic acid and protein, and are secreted into the extracellular environment^26,27^. Furthermore, some reports indicate cell-free miRNAs are stable not only within exosomes, but also in a complex with RNA-binding proteins which include Ago2^28–30^. One recent study has reported that endogenous *miR-92b-3p* is associated with the RNA-induced silencing complex including Ago2 protein, although this study did not evaluate miRNA within exosomal fractions^31^. In the present study, we demonstrated that cell-free *miR-92b-3p* is stable and contained within exosome fractions, rather than bound to Ago2. These results were supported by the presence of *SS18-SSX* within exosomes, confirming a recent report showing that exosomes derived from SS harbor the *SS18-SSX* fusion gene^32^. Importantly, exosomal miRNAs have been suggested to play important roles in intercellular communication^23,33^. We hypothesize that cell-free *miR-92b-3p* contributes to cell-cell communication, resulting in SS progression, and which we will next investigate.

The origin of SS is not clear, despite the name of this tumour. Previous reports have suggested the possibility of a neuroectodermal origin, by use of genome-wide analysis of gene expression in SS tissues using a cDNA microarray^34^. Further, the *miR-92b-3p* inhibitor has been shown to promote glioma cell apoptosis, by targeting Dkk3 and blocking the Wnt/beta-catenin signaling pathway^35^. *miR-92b-3p* has also been reported to be specifically overexpressed in primary brain tumours^36^ and to regulate the development of intermediate cortical progenitors in embryonic mouse brain^37^. Therefore, our results demonstrating that *miR-92b-3p* is abundantly secreted from SS cells also support the possibility that this tumour is of neuroectodermal origin.

In conclusion, the potential clinical significance of serum *miR-92b-3p* for tumour monitoring of SS was demonstrated through use of experimental procedures and a validation study based on independent patient cohorts. Although further studies in large patient cohorts would determine the significance of serum *miR-92b-3p* as a non-invasive biomarker of SS, this methodology could also be a novel approach to detect other soft tissue sarcomas that lack useful circulating biomarkers, and help clinicians to determine treatment strategies.

## Materials and Methods

### Serum Collection

The Institutional Review Board of Okayama University Hospital, National Cancer Center Hospital, Kochi Health Sciences Center and Chiba Cancer Center Hospital approved this study protocol. Written informed consent was obtained from all patients after study approval. All experimental methods were carried out in accordance with relevant guidelines and regulations. Whole blood samples were obtained from patients with SS, alveolar soft part sarcoma, clear cell sarcoma, dedifferentiated liposarcoma, leiomyosarcoma, malignant peripheral nerve sheath tumour, myxofibrosarcoma, solitary fibrous tumour, undifferentiated pleomorphic sarcoma, age-matched benign soft tissue tumour, and healthy individuals at the three major sarcoma institutes in Japan; Okayama University hospital, National Cancer Center Hospital, and Chiba Cancer Center Hospital. These blood samples were obtained at the time of diagnosis, post-operative, post-chemotherapeutic status, or disease progression stage. Murine blood was obtained by cardiac puncture at the indicated time points. Sera were fractionated from whole blood samples by centrifugation at 3,500 rpm for 15 min at 4 °C. The collected serum was centrifuged at 20,000 × g for 15 min at 4 °C, and supernatants were collected and passed through a 0.22-µm-pore filter (Merck Millipore, Billerica, MA, USA), then stored at –80 °C.

### Cell lines and cell culture

Four human SS cell lines, SYO-1, HS-SY-II, YaFuSS, and Yamato-SS were used in this study. SYO-1 was previously established in our laboratory^38^. YaFuSS, HS-SY-II, and Yamato-SS were kindly provided by J. Toguchida, H. Sonobe, and N. Naka^39,40^. The human myxofibrosarcoma cell line NMFH-1 was generously provided by A. Ogose^41^. The human undifferentiated pleomorphic sarcoma cell line UPS2023 was established in our laboratory. Human mesenchymal stem cells (hMSC) were purchased from Lonza (Walkersville, USA). The human human fibrosarcoma cell line HT1080 was purchased from the American Type Culture Collection (ATCC, Manassas, VA, USA). A human malignant peripheral nerve sheath tumour cell line NMS2 was available from the cell bank of RIKEN BioResource Center (Ibaraki, Japan). Cell lines were cultured in Dulbecco’s modified Eagle’s medium (DMEM, Gibco Laboratories, Grand Island, NY) or Roswell Park Memorial Institute media (RPMI, Gibco)-1640 or MSC-BM (Invitrogen, Carlsbad, CA) supplemented with 10 or 15% fetal bovine serum (FBS, Hyclone), 100 units/ml of penicillin G and 100 µg/ml of streptomycin (NACALAI TESQUE, Inc., Kyoto, Japan). Cells were incubated at 37 °C in a humidified atmosphere containing 5% CO_2_.

### Preparation of conditioned medium

The conditioned medium (CM) was changed to FBS-free CM at 24 hours after seeding of cells, and then collected at 24 hours after CM exchange. Collected culture medium was centrifuged at 3,500 rpm for 15 min at 4 °C. The CM supernatant was collected and centrifuged at 20,000 × g for 15 min at 4 °C, and supernatants were collected and passed through a 0.22-µm-pore filter (Merck Millipore), then stored at −80 °C.

### Animal experiments

Animal experiments were performed in accordance with the Animal Care and Use Committee, Okayama University. All animal studies were approved by this committee. BALB/c nu/nu female mice were purchased from CLEA Japan Inc. (Tokyo, Japan) at 4 weeks of age, and given at least 1 week to adapt to their new environment prior to tumour transplantation in a specific-pathogen-free environment. On day 0, the mice were anesthetized with 2% isoflurane, and transplanted in their right hind-quarters with SYO-1 cells (5 × 10^6^ cells/mouse in 100 µL total volume with DMEM suspension). Tumour growth was monitored once each week. Tumour resection was performed 3 weeks after transplantation. Blood samples were taken by cardiac puncture and collected into CAPIJECT^®^ micro collection tubes (TERUMO, Tokyo, Japan) under anesthesia with isoflurane.

### miRNA array

Whole circulating miRNA profiling was performed using a miRNA microarray manufactured by Agilent Technologies (Tokyo, Japan). Two nanograms of extracted RNA were used for each microarray experiment. The results of miRNA microarray analysis were processed using Agilent Feature Extraction software (v10.7.3.1) and analyzed using GeneSpring 12.6.1 software (Agilent Technologies, Tokyo, Japan).

### RNA extraction and RT-qPCR analysis

Total RNA was isolated from cells collected after 24-hrs cell culture using miRNeasy mini Kits (Qiagen, Valencia, CA, USA) according to manufacturer’s instructions. For serum samples and culture media, total RNA was extracted from 200 µL of serum supernatant or concentrated medium using the same extraction kits. RNA samples were reverse transcribed using the TaqMan MicroRNA Reverse Transcription Kit (Applied Biosystems, Foster City, CA, USA). The products were mixed with 5.0 µL of TaqMan 2 × Universal PCR Master mix and 0.50 µL of each primer for qPCR using Agilent Mx3000 P (Agilent Technologies, Santa Clara, CA, USA) instrumentation. Data obtained from RT-qPCR were analyzed using the 2^−∆∆Ct^ method^42^. The miRNA expression levels were normalized using *cel-miR-39* for serum and culture media, and *RNU6B* for tumour cells. Endogenous *miR-16* was used as the normalizer for circulating miRNA quantification. Differences between the groups are presented as ΔCt, indicating differences between Ct values of miRNAs of interest and Ct values of normalizer miRNAs.

### Exosomes isolation from cell culture medium

Exosomes were purified from the culture medium supernatant as previously reported^43^ with partial modification. Each cell line was grown to 60–70% confluence, and then CM was exchanged to FBS-free. The CM samples were collected 24-hrs after medium exchange. The CM was centrifuged at 3,500 rpm for 15 min at 4 °C, followed by further centrifugation at 9,000 × g for 30 min at 4 °C and supernatant was passed through a 0.22-µm-pore filter (Merck Millipore) to remove apoptic bodies, microvesicles, and cell debris. The collected CM supernatant was concentrated to approximately 1 ml using 100 kDa MWCO ultrafiltration membranes (Fisher Scientific, Loughborough, UK) at 4 °C. The sample was then ultracentrifuged (Optima TL-100 (Beckman Coulter, Fullerton, CA, USA) at 100,000 g for 70 min at 4 °C). The resulting pellet was rinsed with PBS, followed by further ultracentrifugation at 100,000 × g for 70 min at 4 °C. Finally, the supernatant was discarded, with exosomes concentrated in the pellet. The obtained exosomes were authenticated by scanning electron microscopy (SEM) and by NS300 Nanosight^®^ nanoparticle analyzer (Malvern Instruments Ltd. Worcestershire, UK).

### Exosomes isolation from human serum

Exosomes were purified from human serum samples by size exclusion chromatography on drip using EV-second^®^ (GL sciences, Tokyo, Japan) in a low-temperature environment. The column was initially equilibrated with 700 µl of PBS twice, followed by a blocking step using 700 µl of FBS. After repeating the wash steps six times with 700 µl of PBS, 200 µl of the collected human serum sample was loaded onto this column followed by collection of 12 consecutive fractions in 100 µl of PBS. CD9 expression in these fractions was analyzed by western blotting and CD9-positive fractions were recognized as the exosome-rich portion^44^.

### Immunoprecipitation and immuno-blot analysis

Immunoprecipitation (IP) for analytical separation of Ago2 from patient serum samples was performed using Protein G Sepharose 4 Fast Flow^®^ (GE Healthcare, Amersham, UK) with anti-Ago2 monoclonal IgG antibody (Wako, Osaka, Japan) according to the product manual. Total protein from cells (10 µg) and exosomes (1 µg) was fractionated using an electrophoretic gradient across Mini-PROTEAN^®^ tris-glycine extended gels (BIO-RAD, Richmond, CA, USA). Loading samples were normalized according to protein concentrations quantified using the Bradford assay^45^. The gels were then transferred onto the Immun-Blot^®^ PVDF membrane (BIO-RAD) under wet electrophoretic conditions. The blotted protein was blocked for 1 hr at room temperature with Odyssey^®^ blocking buffer in PBS (LI-COR, Lincoln, NE, USA) and was followed by incubation overnight at 4 °C with the following primary antibodies: 1:1000 anti-CD9 mouse monoclonal antibody (Abcam, Cambridge, MA, USA); 1:1000 anti-cytochrome-c mouse monoclonal antibody (Abcam); and 1:10000 anti-β-actin mouse monoclonal antibody (Sigma-Aldrich, St. Louis, MO, USA). Thereafter, IRDye^®^ 800CW anti-rabbit IgG and IRDye^®^ 680RD anti-mouse IgG secondary antibodies (LI-COR) were incubated with the protein-blotted membrane for 1 hr at room temperature. Fluorescence was then detected on the Odyssey^®^ imaging system (LI-COR).

### Statistical Analysis

Results were depicted as the mean ± standard deviation or the median with a 25–75% range. Differences in patient demographics and clinical characteristics were measured by the unpaired *t*-test. Statistical differences in quantified miRNA levels were determined by unpaired *t*-test or Analysis of Variance (ANOVA) followed by Holm-Sidak’s multiple comparisons test. Correlations between miRNA and tumour size in animal experiments were assessed with Pearson’s correlation coefficient. ROC curve analysis was performed to examine the diagnostic potential of serum miRNA expression levels. A two-sided *p*-value of less than 0.05 was considered statistically significant. Statistical analysis was carried out using GraphPad Prism version 6.0 h (GraphPad Software, San Diego, CA, USA) and R (version 3.3.1).

### Data Availability

All data generated or analyzed during this study are included in this published article and its Supplementary Information files.

## Electronic supplementary material

Supplementary information
