# Supplementary material for: Circulating MicroRNA-92b-3p as a Novel Biomarker for Monitoring of Synovial Sarcoma
Source: Sci Rep. 2017 Nov 7;7:14634. doi: 10.1038/s41598-017-12660-5 (PMC5676745; doi:10.1038/s41598-017-12660-5)
Supplement: Supplementary file 1 — Supplementary information [file 41598_2017_12660_MOESM1_ESM.pdf]

Supplementary information

Circulating *MicroRNA-92b-3p* as a Novel Biomarker for Monitoring of Synovial Sarcoma

Koji Uotani[1], Tomohiro Fujiwara[1,2], Aki Yoshida[1], Shintaro Iwata[3], Takuya Morita[1], Masahiro Kiyono[1], Suguru Yokoo[1], Toshiyuki Kunisada[4], Ken Takeda[5], Joe Hasei[1], Kunihiko Numoto[6], Yutaka Nezu[7], Tsukasa Yonemoto[3], Takeshi Ishii[3], Akira Kawai[8], Takahiro Ochiya[7], Toshifumi Ozaki[1]

[1]Department of Orthopaedic Surgery, Okayama University Graduate School of Medicine, Dentistry, and Pharmaceutical Sciences
[2]Center of Innovative Medicine, Okayama University Hospital
[3]Department of Orthopaedic Surgery, Chiba Cancer Center
[4]Department of Medical Materials for Musculoskeletal Reconstruction, Okayama University Graduate School of Medicine, Dentistry, and Pharmaceutical Sciences
[5]Department of Intelligent Orthopaedic System, Okayama University Graduate School of Medicine, Dentistry, and Pharmaceutical Sciences
[6]Department of Orthopaedic Surgery, Kochi Health Sciences Center
[7]Division of Molecular and Cellular Medicine, National Cancer Center Research Institute
[8]Department of Musculoskeletal Oncology, National Cancer Center Hospital

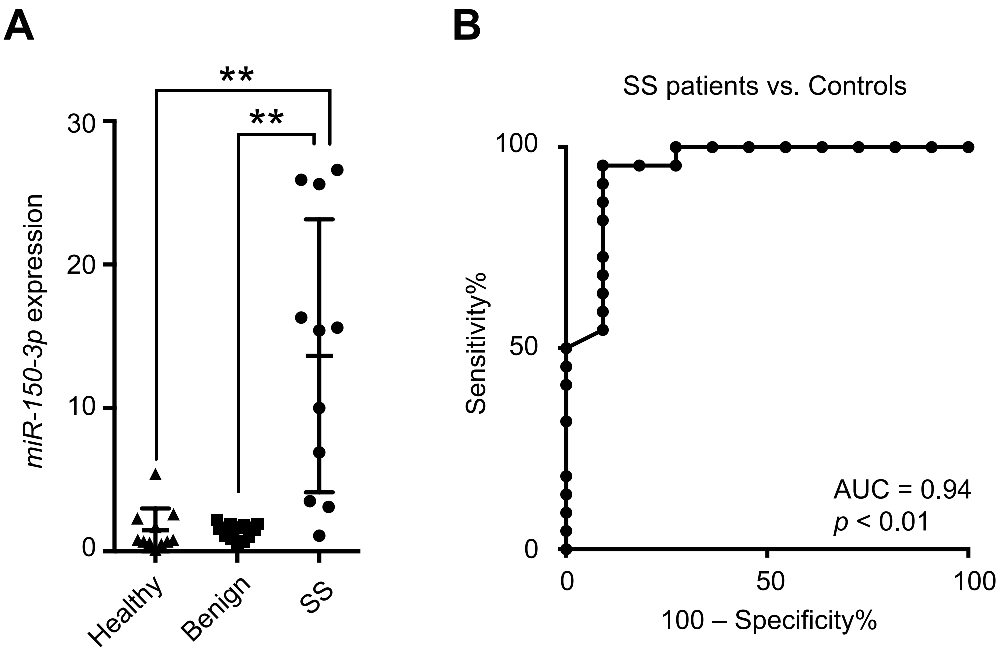

Supplementary Figure S1. Serum *miR-150-3p* expression levels in the validation cohort. (A) Serum *miR-150-3p* expression levels in SS patients, age-matched benign tumor patients, and control individuals in the validation cohort ($p < 0.01$). **$p < 0.01$; one-way ANOVA with Holm-Sidak's multiple comparison test. (B) Receiver-operating characteristics curve analysis indicated the AUC of 0.94 (95% confidence interval = 0.86 – 1.0)

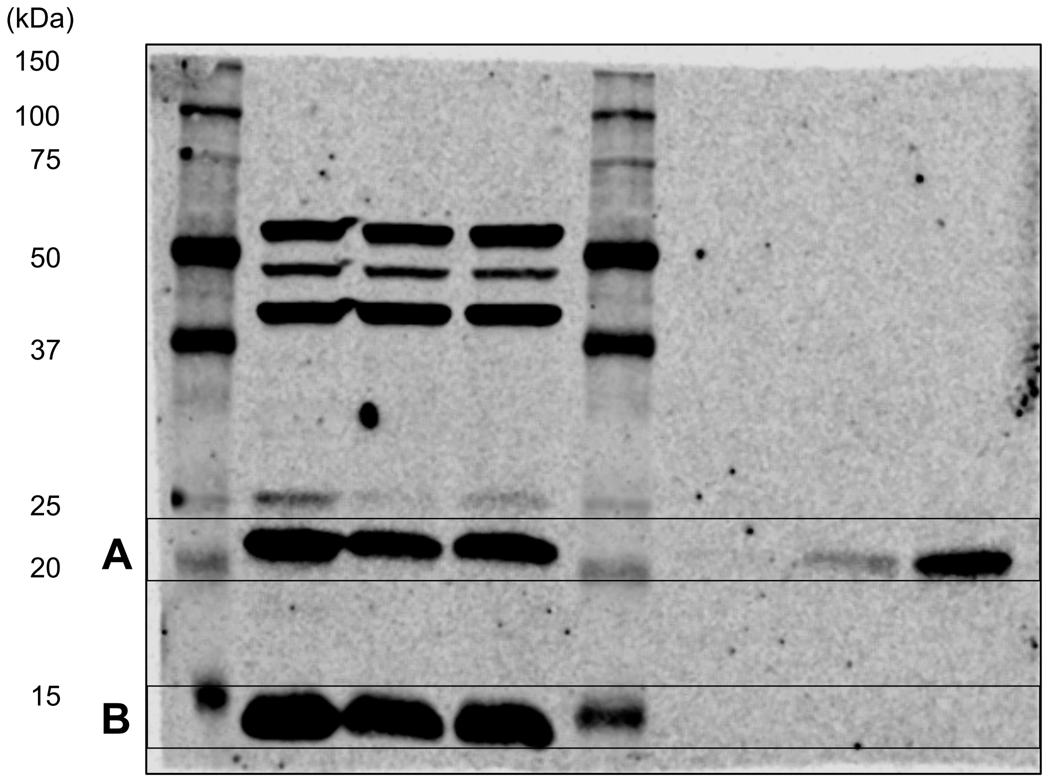

Supplementary Figure S2. Full-length blots shown in Figure 5C. (A) CD9. (B) Cytochrome-C.

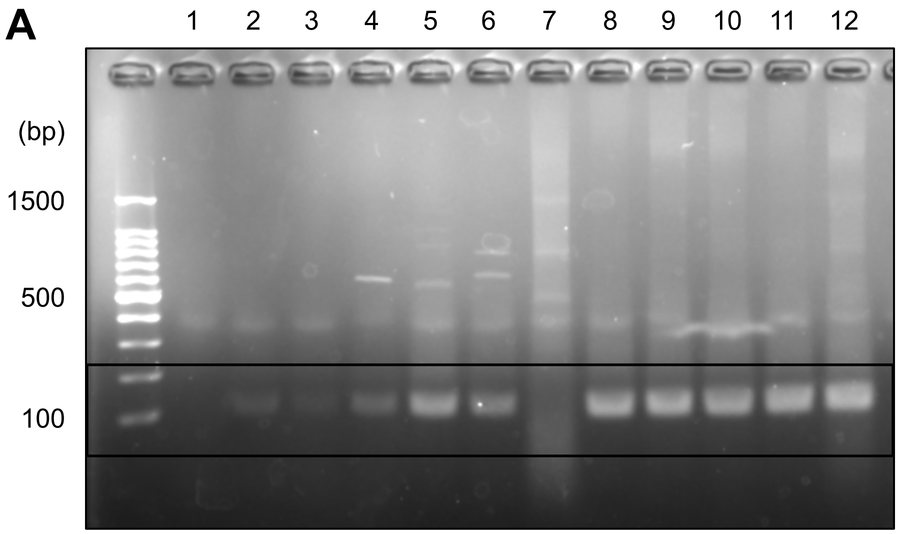

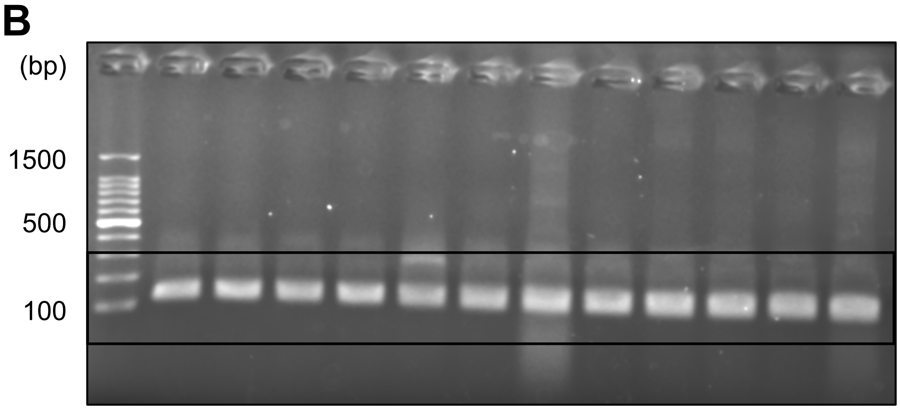

Lane No.	Type of samples	Cell lines
1	Exosome	HT1080
2		SYO-1
3		HS-SY-II
4		YaFuSS
5		Yamato-SS
6		Aska-SS
7	Cells	HT1080
8		SYO-1
9		HS-SY-II
10		YaFuSS
11		Yamato-SS
12		Aska-SS

Supplementary Figure S3. Full-length gels shown in Figure 5D. (A) *SS18-SSX*. (B) *GAPDH*.

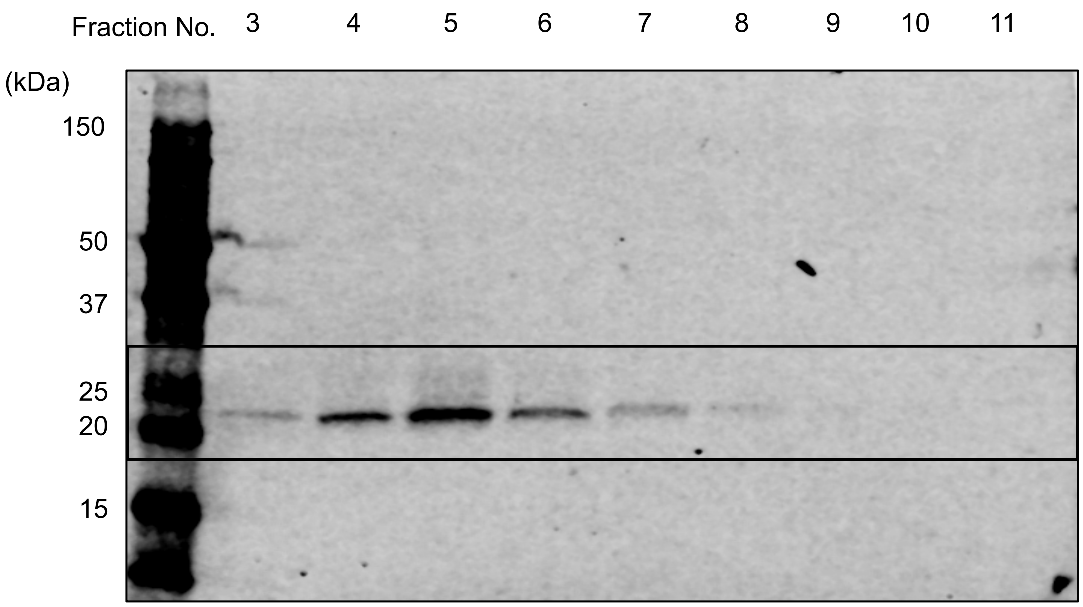

Supplementary Figure S4. Full-length blots for CD9 shown in Figure 5F.

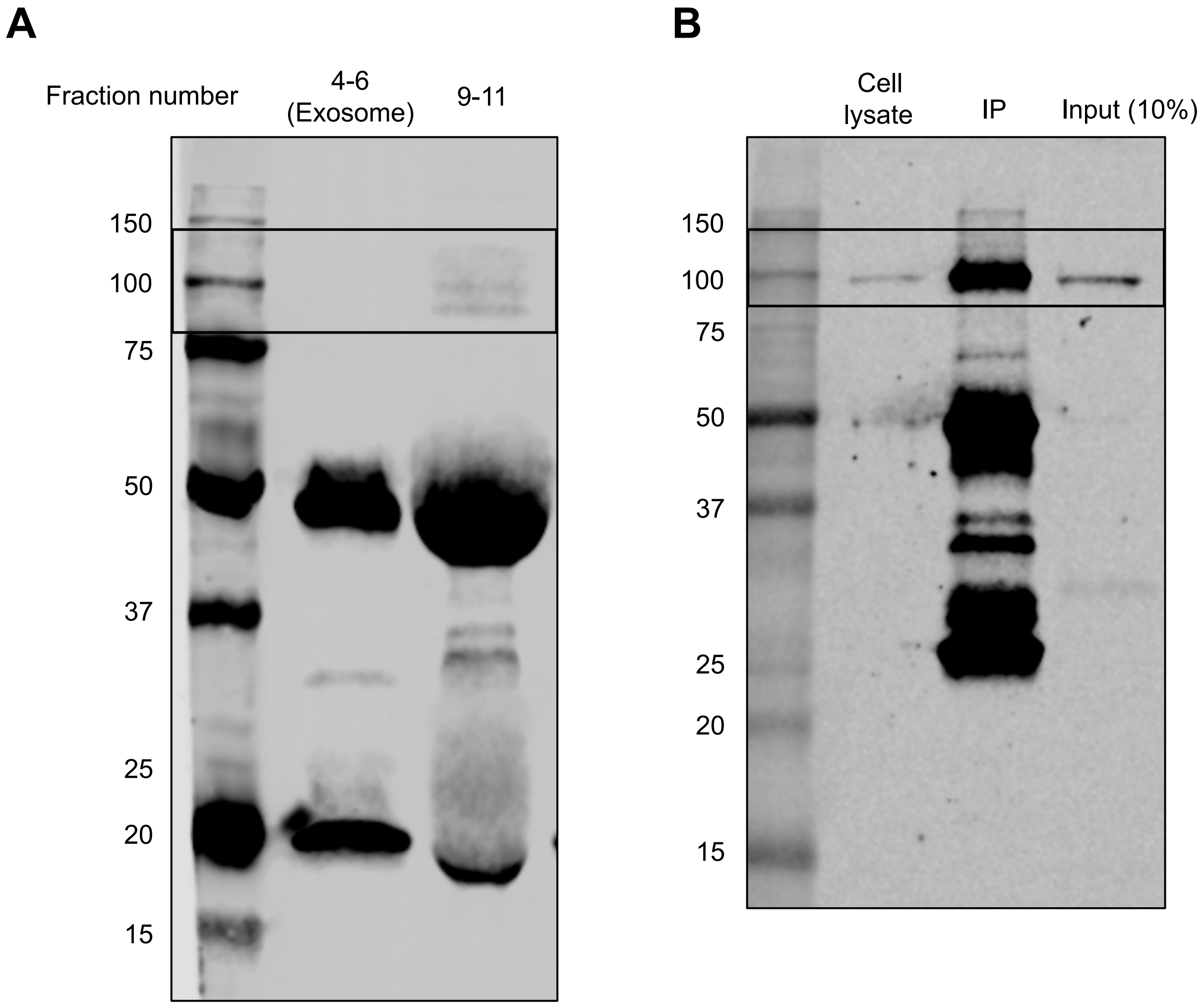

Supplementary Figure S5. Full-length blots shown in Figure 5G. (A) Western blotting of fractions collected by EV-second procedure using a serum sample of patient with synovial sarcoma, which were immunoprecipitated (IP) using human anti-Ago2 monoclonal antibody. (B) Western blotting of SYO-1 cell lysate fractions, which were immunoprecipitated using human anti-Ago2 monoclonal antibody.

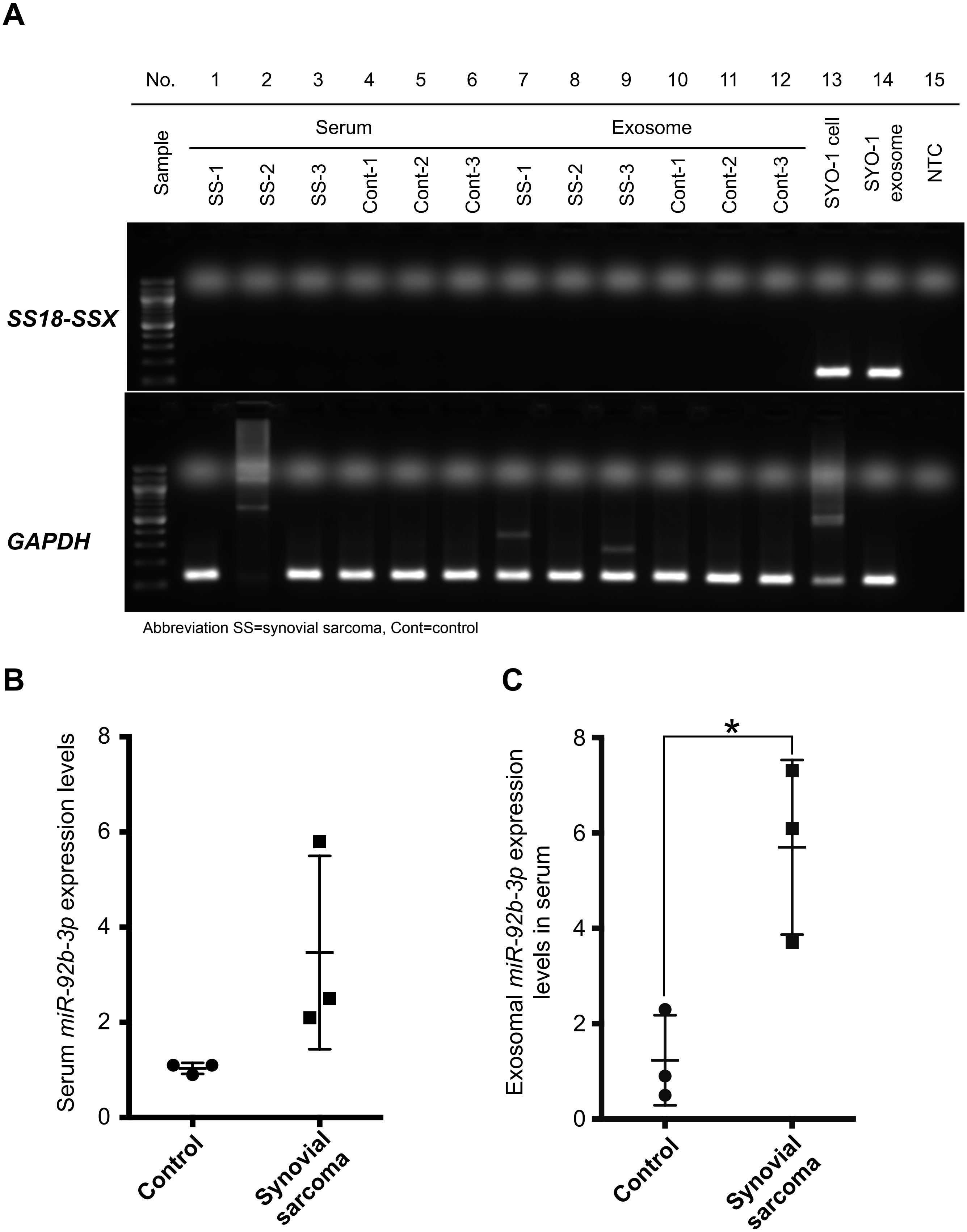

Supplementary Figure S6. *SS18-SSX* expression in exosomes from SS-patient serum. (A) *SS18-SSX* fusion gene transcript by polymerase chain reaction (PCR). *SS18-SSX* was not detectable in either the serum or the exosomes of SS patients and controls (each n = 3). Cell lysate and exosomes of the SYO-1 SS cell line were used as positive controls. Ct values of each sample are presented **in Supplementary Table S5**. The threshold of PCR of *SS18-SSX* is presented in **Supplementary Table S6.** (B) Serum *miR-92b-3p* expression levels in SS-patient serum analyzed in (A). (C) Serum *miR-92b-3p* expression levels in the exosomes derived from SS-patient serum analyzed in (A). *$p < 0.05$; Student's *t*-test.

Supplemental Table S1. Characteristics of patients and healthy individuals in each cohort

Variables		Screening set			Validation set		
		Synovial sarcoma patients (n = 9)	Benign tumor patients (n = 9)	Healthy individuals (n = 9)	Synovial sarcoma patients (n = 12)	Benign tumor patients (n = 12)	Healthy individuals (n = 12)
Age (years)[a]	Median (range)	28 (21-56)	35 (25-56)	35 (26-58)	43.5 (11-71)	43.5 (11-68)	54.5 (29-79)
Gender	Male	3	3	4	6	5	5
	Female	6	6	5	6	7	7

[a]Age at diagnosis

Supplemental Table S2. Clinical characteristics of synovial sarcoma patients in each cohort

Variables	Discovery cohort (n = 9)	Validation cohort (n = 12)
Age (years)[a]		
Median (range)	28 (21-56)	43.5 (11-71)
11-20	-	5
21+	9	7
Gender		
Male	3	6
Female	6	6
Location		
Extremities, %	55.6	66.7
Body trunks, %	44.4	33.3
Upper limb	2	-
Lower limb	3	8
Body trunks	4	4
Size of the lesion, mm		
Median (range)	9.2 (4-60)	8.5 (4-15)
Metastasis at diagnosis		
Present	2	3
Absent	7	9
Histological subtype		
Monophasic	5	8
Biphasic	3	4
Treatment		
C+OP	7	10
C+OP+R	1	-
C+R	-	1
C	-	1
OP	1	-
Neoadjuvant chemotherapy		
DOX+IFO	8	2
DOX	-	1
GEM+DOC	-	1
Other	-	8
Disease status		
CDF	-	6
NED	6	1
AWD	2	1
DOD	1	4

Abbreviations C=chemotherapy, OP=operative surgery, R=radiation, DOX=doxorubicin, IFO=ifosfamide, GEM=gemcitabine, CDF=continuous disease free, NED=no evidence of disease, AWD=alive with disease, DOD=died of disease

[a]Age at diagnosis

Supplemental Table S3. Clinical correlation of *miR-92b-3p* and *miR-150-3p* expression levels in serum from synovial sarcoma patients

Variables	Serum *miR-92b-3p* levels mean ± SD (N = 12)	*P*	Serum *miR-150-3p* levels mean ± SD (N = 12)	*P*
Age (years)[a]				
≤ 43.5	0.93 ± 0.99 (n = 6)	0.093	9.8 ± 7.4 (n = 6)	0.31
> 43.5	3.8 ± 4.1 (n = 6)		14 ± 5.1 (n = 6)	
Gender				
Male	1.2 ± 0.92 (n = 6)	0.48	13 ± 6.2 (n = 6)	0.70
Female	3.5 ± 4.4 (n = 6)		11 ± 7.0 (n = 6)	
Location				
Extremities	3.2 ± 3.8 (n = 8)	0.15	13 ± 5.0 (n = 8)	0.28
Trunks	0.77 ± 0.51 (n = 4)		8.6 ± 8.5 (n = 4)	
Tumor size, cm				
≤ 8.5	1.3 ± 1.7 (n = 6)	0.093	8.9 ± 6.7 (n = 6)	0.13
> 8.5	3.5 ± 4.1 (n = 6)		15 ± 4.9 (n = 6)	
Lung metastasis				
Negative	2.4 ± 3.6 (n = 9)	0.73	13 ± 6.7 (n = 9)	0.60
Positive	2.3 ± 2.2 (n = 3)		9.8 ± 6.2 (n = 3)	

[a]Age at diagnosis

Supplementary Table S4. Patients demographics of the soft tissue sarcomas other than SS

Case No.	Age (years)[a]	Gender	Diagnosis
#1	19	F	Alveolar soft part sarcoma
#2	22	F	Alveolar soft part sarcoma
#3	52	M	Clear cell sarcoma
#4	73	M	Dedifferentiated liposarcoma
#5	62	F	Dedifferentiated liposarcoma
#6	64	F	Leiomyosarcoma
#7	57	F	Leiomyosarcoma
#8	29	M	Malignant peripheral nerve sheath tumor
#9	49	F	Malignant peripheral nerve sheath tumor
#10	54	F	Malignant peripheral nerve sheath tumor
#11	60	M	Malignant peripheral nerve sheath tumor
#12	28	F	Malignant peripheral nerve sheath tumor
#13	23	F	Malignant peripheral nerve sheath tumor
#14	55	F	Myxofibrosarcoma
#15	59	F	Myxofibrosarcoma
#16	67	M	Myxofibrosarcoma
#17	49	M	Myxoid liposarcoma
#18	21	M	Myxoid liposarcoma
#19	52	F	Solitary fibrous tumor
#20	23	M	Undifferentiated pleomorphic sarcoma
#21	42	M	Undifferentiated pleomorphic sarcoma
#22	63	F	Undifferentiated pleomorphic sarcoma
#23	73	F	Undifferentiated pleomorphic sarcoma
#24	83	M	Undifferentiated pleomorphic sarcoma

[a]Age at diagnosis

Supplementary Table S5. Ct values of *GAPDH* and *SS18-SSX* for each sample.

Lane no.	1	2	3	4	5	6	7	8	9	10	11	12	13	14	15
Sample	Serum						Exosome						SYO-1 cell	SYO-1 exosome	NTC
	SS-1	SS-2	SS-3	Cont-1	Cont-2	Cont-3	SS-1	SS-2	SS-3	Cont-1	Cont-2	Cont-3			
GAPDH	34.41	33.97	33.41	32.97	34.19	33.74	36.78	35.89	33.92	34.87	35.29	35.53	23.47	29.73	No Ct
SS18-SSX	No Ct	No Ct	No Ct	No Ct	No Ct	No Ct	No Ct	No Ct	No Ct	No Ct	No Ct	No Ct	28.65	35.57	No Ct

Abbreviation SS=synovial sarcoma, Cont=control

Supplementary Table S6. Threshold for detecting ***SS18-SSX*** by PCR

Amount of RNA of SYO-1 SS cells	***GAPDH***	***SS18-SSX***
1 pg	56.98	No Ct
10 pg	53.72	No Ct
100 pg	44.99	49.54
1 ng	39.43	43.9
10 ng	34.66	38.94
100 ng	29.5	33.97
500 ng	26.11	30.66
NTC	54.98	No Ct

Supplementary Table S7. Summary of miRNA dysregulations in synovial sarcoma cells and tissues that have been previously reported

Upregulated miRNAs in synovial sarcoma cells/tissues		
miR-9-5p[21]	*miR-193a-5p*[23]	*miR-379*[23]
miR-17-5p[24,26]	*miR-199a*[24]*-3p*[21, 23]<br>*miR-199b-3p*[21]	*miR-381*[23]
miR-96[22]	*miR-200a*[22],*b*[22,24]*-3p*[21]<br>*miR-200c*[21, 24]	*miR-429*[22]
miR-98[23]	*miR-203*[22]	*miR-411*[23]
miR-99a, b[23]	*miR-214*[23, 24]	*miR-495*[22]
miR-125a-3p,b1,2[23]	*miR-299-39*[23]	*miR-574-3p*[23]
miR-127[18]*-3p*[23]	*miR-337-5p*[23]	*miR-668*[22]
miR-141[24]	*miR-368*[24]	*miR-874*[23]
miR-182[22]	*miR-375*[22]	*miR-1468*[22]
miR-183[22, 24, 25]	*miR-376a*[23,24]*-3p*[21]<br>*miR-376c*[21, 23]	*let-7e,i*[23]
Downregulated miRNAs in synovial sarcoma cells/tissues		
miR-15b[24]	*miR-145*[22, 24] *-5p*[21]	*miR-542-3p*[16]*,5p*[22]
miR-18b[23]	*miR-146b*[24]	*miR-548j*[22]
miR-20a[23]*,b*[24]	*miR-150-5p*[21]	*miR-550*[22]
miR-21[21, 24]	*miR-221*[22]	*miR-612*[23]
miR-23a,b[24]	*miR-222*[22, 24]	*miR-618*[22]
miR-24[24]	*miR-223*[21, 24]	*miR-636*[23]
miR-27a,b[24]	*miR-335*[24]	*miR-638*[23]
miR-29a,b,c[24]	*miR-338-5p*[22]	*miR-663*[23]
miR-30a-5p,d,e-5p[24]	*miR-339-5p*[22]	*miR-675*[23]
miR-34b[22]*,c-3p*[22]*,5p*[22]	*miR-346*[23]	*miR-1225-3p*[23]
miR-106a[24]	*miR-378a-3p,i,g*[21]	*miR-1226*[23]
miR-126[18]	*miR-424*[22, 24]	*miR-1233*[23]
miR-133b[21]	*miR-450a*[22]	*miR-1234*[23]
miR-139-3p[23]	*miR-451*[24]	*let-7a*[23]*,F*[24]
miR-142-3p[22]*,-5p*[22]	*miR-503*[22]	
miR-143[24]	*miR-511*[22]	
